# Adolescent Psychedelic Use and Psychotic or Manic Symptoms

**DOI:** 10.1001/jamapsychiatry.2024.0047

**Published:** 2024-03-13

**Authors:** Otto Simonsson, Miriam A. Mosing, Walter Osika, Fredrik Ullén, Henrik Larsson, Yi Lu, Laura W. Wesseldijk

**Affiliations:** 1 Department of Neurobiology, Care Sciences and Society, Karolinska Institutet, Stockholm, Sweden; 2 Department of Neuroscience, Karolinska Institutet, Stockholm, Sweden; 3 Department of Cognitive Neuropsychology, Max Planck Institute for Empirical Aesthetics, Frankfurt am Main, Germany; 4 Melbourne School of Psychological Sciences, Faculty of Medicine, Dentistry, and Health Sciences, University of Melbourne, Melbourne, Victoria, Australia; 5 Department of Medical Epidemiology and Biostatistics, Karolinska Institutet, Stockholm, Sweden; 6 Stockholm Health Care Services, Southern Stockholm Psychiatric District, Region Stockholm, Stockholm, Sweden; 7 School of Medical Sciences, Örebro University, Örebro, Sweden; 8 Department of Psychiatry, Amsterdam University Medical Center, University of Amsterdam, Amsterdam, the Netherlands

## Abstract

**Importance:**

While psychedelic-assisted therapy has shown promise in the treatment of certain psychiatric disorders, little is known about the potential risk of psychotic or manic symptoms following naturalistic psychedelic use, especially among adolescents.

**Objective:**

To investigate associations between naturalistic psychedelic use and self-reported psychotic or manic symptoms in adolescents using a genetically informative design.

**Design, Setting, and Participants:**

This study included a large sample of adolescent twins (assessed at age 15, 18, and 24 years) born between July 1992 and December 2005 from the Swedish Twin Registry and cross-sectionally evaluated the associations between past psychedelic use and psychotic or manic symptoms at age 15 years. Individuals were included if they answered questions related to past use of psychedelics. Data were analyzed from October 2022 to November 2023.

**Main Outcomes and Measures:**

Primary outcome measures were self-reported psychotic and manic symptoms at age 15 years. Lifetime use of psychedelics and other drugs was also assessed at the same time point.

**Results:**

Among the 16 255 participants included in the analyses, 8889 were female and 7366 were male. Among them, 541 participants reported past use of psychedelics, most of whom (535 of 541 [99%]) also reported past use of other drugs (ie, cannabis, stimulants, sedatives, opioids, inhalants, or performance enhancers). When adjusting for substance-specific and substance-aggregated drug use, psychedelic use was associated with reduced psychotic symptoms in both linear regression analyses (β*, *−0.79; 95% CI, −1.18 to −0.41 and β*, *−0.39; 95% CI, −0.50 to −0.27, respectively) and co-twin control analyses (β*, *−0.89; 95% CI, −1.61 to −0.16 and β*, *−0.24; 95% CI, −0.48 to −0.01, respectively). In relation to manic symptoms, likewise adjusting for substance-specific and substance-aggregated drug use, statistically significant interactions were found between psychedelic use and genetic vulnerability to schizophrenia (β*, *0.17; 95% CI, 0.01 to 0.32 and β*, *0.17; 95% CI, 0.02 to 0.32, respectively) or bipolar I disorder (β*, *0.20; 95% CI, 0.04 to 0.36 and β*, *0.17; 95% CI, 0.01 to 0.33, respectively).

**Conclusions and Relevance:**

The findings in this study suggest that, after adjusting for other drug use, naturalistic use of psychedelic may be associated with lower rates of psychotic symptoms among adolescents. At the same time, the association between psychedelic use and manic symptoms seems to be associated with genetic vulnerability to schizophrenia or bipolar I disorder. These findings should be considered in light of the study’s limitations and should therefore be interpreted with caution.

## Key Points

**Question:**

Is there an association between psychedelic use and psychotic or manic symptoms in adolescents?

**Findings:**

In a cross-sectional study of 16 255 adolescent twins, psychedelic use was significantly associated with lower rates of psychotic symptoms when adjusting for other drug use. Psychedelic use was significantly associated with more manic symptoms for individuals with a higher genetic vulnerability to schizophrenia or bipolar I disorder than for individuals with a lower genetic vulnerability.

**Meaning:**

The findings suggest that psychedelic use may be associated with lower rates of psychotic symptoms but the association between psychedelic use and manic symptoms seems to be associated with genetic vulnerability.

## Teaser

This cross-sectional study evaluates the association between adolescent psychedelic use and self-reported psychotic or manic symptoms.

## Introduction

Recently, the use of lysergic acid diethylamide (LSD) has increased among adolescents in the US.^1^ While there is evidence that LSD and other 5HT2AR agonist psychedelics such as psilocybin may be relatively safe in controlled settings,^2^ less is known about the risks associated with naturalistic use of psychedelics, especially among adolescents. Hence, there is a pressing need for studies on the possible effects of psychedelic use among adolescents to inform both harm reduction efforts and future research.

Guidelines on psychedelic research recommend that individuals with a personal or family history of psychotic or bipolar disorders be excluded from participation in clinical trials.^3^ There are concerns that a personal or family history of these conditions may elevate the risk of psychotic or manic episodes following administration of psychedelics, but exclusion of such individuals from clinical trials of psychedelic-assisted therapy also limits the possibility of quantifying psychiatric risks.^4,5^ Apart from recent clinical research^6,7^ on psilocybin-assisted therapy in patients with bipolar II disorder, available empirical evidence is mainly based on research designs prone to familial confounding and similar biases.^8,9,10,11^ It is therefore important to use genetically informative approaches to better understand the nature and causal underpinnings of observed associations.

While twin data have been used to examine the association between psychiatric problems and cannabis use,^12^ twin modeling studies have rarely been used in psychedelic research. By using twin research designs, it is possible to control for familial confounding and test whether the association between psychedelic use and psychotic or manic symptoms is in line with a causal hypothesis.^13^ For example, finding that pairs of monozygotic twins who differ in psychedelic use do not differ significantly in psychotic or manic symptoms would suggest that the association is likely not causal and is instead due to familial (genetic and shared environmental) confounding. However, the effects of psychedelic use could also depend on an individual’s genotype (gene-environment interaction). For instance, individuals with a high genetic susceptibility to certain psychotic or bipolar disorders may experience more psychotic or manic symptoms after using psychedelics compared to those with a low genetic risk for these conditions. This could mask associations and cause mixed findings.

Here, we used a large genetically informative sample of twins to investigate associations between psychedelic use and psychotic or manic symptoms, the role of familial confounding in such associations (co-twin control modeling), and whether the risk of psychotic or manic symptoms depends on interactions between psychedelic use and genetic vulnerability to schizophrenia or bipolar I disorder.

## Methods

### Study Population

The Child and Adolescent Twin Study in Sweden (CATSS), conducted by the Swedish Twin Registry, includes data on Swedish twins who—together with their parents—were invited to participate for the first time at age 9 years. Follow-up assessments were conducted when the twins were 15, 18, and 24 years. At age 15 years, 16 255 twins answered questions about past use of LSD or psilocybin. Among these, 15 862 and 15 717 answered questions about psychotic and manic symptoms, respectively. At a later stage, 9426 of these twins also provided genotype data.^14^ Ethical approval for this study was granted by the Swedish Ethical Review Authority. Written informed consent was obtained from all participants. Twins provided consent for twin questionnaires from age 15 years. Parents provided consent for parent questionnaires. The study followed the Strengthening the Reporting of Observational Studies in Epidemiology (STROBE) reporting guideline. Data were analyzed from October 2022 to November 2023.

### Measures

#### Drug Use

Twins were asked about past use of various drugs at age 15 years. Twins who indicated that they had never used LSD or psilocybin were coded as 0 for psychedelic use, while those who indicated that they had used LSD or psilocybin were coded as 1. Other drug use items were recoded into separate categories based on the type of drug: alcohol, tobacco, cannabis, stimulants, sedatives, opioids, inhalants, and performance enhancers, resulting in 8 binary (yes/no) variables (eTable 1 in Supplement 1). Unspecific responses (eg, “don’t know” and “don’t want to answer”) were coded as missing values.

#### Psychotic and Manic Symptoms

Self-reported psychotic and manic symptoms were assessed at age 15 years. Self-reported psychotic symptoms were assessed with a 7-item questionnaire that broadly corresponded with the Adolescent Psychotic-Like Symptom Screener^15^ (eTable 2 in Supplement 1). The items (eg, “other people read my thoughts”) were assessed on a 3-point scale (0 = “never,” 1 = “maybe,” 2 = “definitely”), with summed total scores ranging from 0 to 14. Self-reported manic symptoms were assessed using the 10-item Child Mania Rating Scale Brief Version^16^ (eTable 3 in Supplement 1). The items (eg, “have periods of too much energy”) were assessed on a 4-point scale (0 = “never/rarely,” 1 = “sometimes,” 2 = “often,” 3 = “very often”), with summed total scores ranging from 0 to 30.

The parents of the twins were asked to rate them on psychotic and manic symptoms at age 15 years, but there were approximately 19% fewer parent-reported than self-reported responses. There were also no follow-up questions about psychedelic use at age 18 and 24 years. We therefore focused the analyses on self-reported data at age 15 years, but supplementary analyses were still conducted with parent-reported psychotic and manic symptoms at age 15 years and self- and parent-reported psychotic and manic symptoms at older ages (18 and 24 years).

#### Genetic Risk

We estimated genetic risk using polygenic scores, which are a weighted count of risk alleles multiplied by each genetic variant’s effect size on a phenotype, as identified in earlier genome-wide association studies.^17^ We calculated polygenic scores for schizophrenia and bipolar I disorder using summary statistics from genome-wide association studies of schizophrenia^18^ and bipolar I disorder.^19^ CATSS study samples were independent of these genome-wide association study meta-analyses. Each discovery genome-wide association study results file was filtered based on available information for minor allele frequency (>0.01) and imputation quality (>0.9). Insertion-deletions, as well as asymmetric or ambiguous (AT, TA, CG, GC), multiallelic and duplicate position single-nucleotide variants were excluded. We used SBayesR version 2.04.3^20^ to rescale the summary statistics, using the banded sparse matrices provided by the authors, and default parameter settings. We then generated the polygenic scores using PLINK2. The polygenic scores were standardized. A principal component analysis was performed in the study sample to generate 20 ancestry covariates that were added to all association analyses that included polygenic scores to adjust for ancestral differences in the sample.

### Statistical Analyses

Both unadjusted and adjusted linear regression models with psychedelic use as the independent variable and psychotic or manic symptoms as the dependent variable were fitted to estimate associations between psychedelic use and psychotic or manic symptoms. Within-pair linear regression models (ie, co-twin control analyses) were fitted to investigate associations free of familial confounding between psychedelic use and psychotic or manic symptoms.^13^ These analyses were conducted within monozygotic twin pairs only. Both unadjusted and adjusted models were estimated. Both unadjusted and adjusted linear regression models were fitted to estimate the interactions between psychedelic use and an individual’s polygenic scores for schizophrenia or bipolar I disorder on psychotic or manic symptoms (ie, whether the association between psychedelic use and psychotic or manic symptoms varied depending on an individual’s genetic vulnerability to schizophrenia or bipolar I disorder).

All analyses were conducted in Stata version 18. All models were fitted, first, unadjusted, with only sex as a covariate (except in co-twin control analyses as identical twins have the same sex) and, second, adjusted for other drug variables as additional covariates. Age was not controlled for since all participants were the same age. Two different drug-adjusted analyses were applied: (1) substance-specific adjusted analyses controlling for sex and past use of alcohol, tobacco, cannabis, stimulants, sedatives, opioids, and inhalants (performance enhancers were dropped from these analyses due to high variance inflation factor >10]; see eTable 4 in Supplement 1) and (2) substance-aggregated adjusted analyses controlling for sex, past use of alcohol or tobacco (collapsed into a single variable), and past use of cannabis, stimulants, sedatives, opioids, inhalants, or performance enhancers (collapsed into a single variable). The 20 principal components (ancestry covariates) were always included as covariates in analyses using polygenic scores. The genotyping platform that was used in different subsamples (ie, Global Screening Array and PsychChip) was also included as a covariate in analyses using polygenic scores. The analyses that investigated interactions with polygenic scores also adjusted for interactions with all the principal components. We controlled for relatedness of the sample by using the robust standard error estimator for clustered observations in Stata.^21^ In any given analysis, if data were missing from any of the variables in the model, these data were excluded from that specific analysis. The scales for psychotic and manic symptoms were standardized. In all tests, a 2-sided *P* < .05 was used as significance threshold.

## Results

### Descriptive Statistics

Among the 16 255 participants included in the analyses, 8889 were female and 7366 were male. Tables 1 and 2 present the descriptive statistics of participants with responses (yes, no) on past use of psychedelics (N = 16 255) and the other drug categories. As shown in the tables, 541 participants (3% of sample) reported past use of psychedelics. Among those who reported psychedelic use, 6 of 541 participants reported no past use of cannabis, stimulants, sedatives, opioids, inhalants, or performance enhancers. There were 119 monozygotic twin pairs discordant for psychedelic use. There was a lower proportion of male participants and also a lower proportion of certain drug use (ie, tobacco, cannabis, stimulants, sedatives, and opioids) in the subsample of participants who provided genotyped data (eTable 5 in Supplement 1).

**Table 1.** Descriptive Statistics^a^

Variable	Past use of psychedelics, No. of cases	
	Yes (n = 541)	No (n = 15 714)
Past use of alcohol		
Yes	533	6810
No	8	8900
Past use of tobacco		
Yes	207	4319
No	332	11 363
Past use of cannabis, stimulants, sedatives, opioids, inhalants, or performance enhancers		
Yes	535	1313
No	6	14 401
Past use of cannabis		
Yes	527	356
No	12	15 342
Past use of stimulants		
Yes	528	60
No	13	15 654
Past use of sedatives		
Yes	507	221
No	28	15 465
Past use of opioids		
Yes	527	664
No	14	15 050
Past use of inhalants		
Yes	509	334
No	28	15 362
Past use of performance enhancers		
Yes	507	9
No	34	15 702
Sex		
Female	226	8663
Male	315	7051
Zygosity		
Monozygotic	144	4693
Monozygotic twin pairs discordant on psychedelic use	119	119

^a^ Due to missing data, total numbers for each category may not add up to total number of responses on past use of psychedelics (N = 16 255).

**Table 2.** Clinical Statistics^a^

Variable	Past use of psychedelics			
	Yes (n = 541)		No (n = 15 714)	
	No. of cases	Mean (SD)	No. of cases	Mean (SD)
Polygenic scores				
Schizophrenia	295	0.00 (1.02)	9131	−0.03 (1.00)
Bipolar I disorder	295	0.01 (0.98)	9131	−0.01 (0.99)
Psychotic symptoms (age 15 y)				
Self-reported psychotic symptoms	486	2.27 (2.50)	14 269	2.15 (2.45)
Parent-reported psychotic symptoms	373	0.34 (0.90)	11 593	0.33 (0.87)
Manic symptoms (age 15 y)				
Self-reported manic symptoms	494	6.16 (4.59)	14 464	4.87 (3.71)
Parent-reported manic symptoms	378	2.16 (2.77)	11 713	1.87 (2.52)

^a^ Standardized scores are presented for polygenic scores. Unstandardized scores are presented for psychotic and manic symptoms. Due to missing data, total numbers for each category may not add up to total number of responses on past use of psychedelics (N = 16 255).

### Regression Models

#### Associations Between Psychedelic Use and Self-Reported Psychotic or Manic Symptoms

Regression model results are presented in Table 3. In unadjusted analyses, psychedelic use was associated with more psychotic and manic symptoms (β, 0.09; 95% CI, 0.00 to 0.18 and β, 0.38; 95% CI, 0.27 to 0.48, respectively). In drug-adjusted analyses, this association was reversed (ie, psychedelic use was associated with fewer psychotic and manic symptoms). A negative association between psychedelic use and psychiatric symptoms was consistent regardless of whether a substance-specific adjustment of drug use was applied (psychotic symptoms: β, −0.79; 95% CI, −1.18 to −0.41 and manic symptoms: β, −1.02; 95% CI, −1.44 to −0.59) or whether drug use was aggregated into 2 independent variables (ie, alcohol or tobacco; all other drugs; psychotic symptoms: β, −0.39; 95% CI, −0.50 to −0.27 and manic symptoms: β, −0.17; 95% CI, −0.30 to −0.05).

**Table 3.** Model Estimates

Model	Unadjusted analyses^a^				Substance-specific adjusted analyses^b^				Substance-aggregated adjusted analyses^c^			
	β (95% CI)	*t* Value (*df*)	*P* value	No. of observations in model	β (95% CI)	*t* Value (*df*)	*P* value	No. of observations in model	β (95% CI)	*t* Value (*df*)	*P* value	No. of observations in model
**Self-reported psychotic symptoms**												
Linear regression	0.09 (0.00 to 0.18)	2.00 (14 752)	.046	14 755	−0.79 (−1.18 to −0.41)	−4.05 (14 662)	<.001	14 672	−0.39 (−0.50 to −0.27)	−6.67 (14 750)	<.001	14 755
Co-twin control	0.02 (−0.17 to 0.22)	0.24 (103)	.81	104	−0.89 (−1.61 to −0.16)	−2.40 (95)	.02	103	−0.24 (−0.48 to −0.01)	−2.06 (101)	.04	104
SCZ GxE	0.00 (−0.12 to 0.11)	−0.08 (8650)	.94	8696	0.00 (−0.12 to 0.12)	−0.01 (8602)	>.99	8655	0.00 (−0.12 to 0.11)	−0.02 (8648)	.99	8696
BIP GxE	0.03 (−0.11 to 0.16)	0.38 (8650)	.70	8696	0.04 (−0.10 to 0.19)	0.61 (8602)	.54	8655	0.03 (−0.10 to 0.16)	0.43 (8648)	.66	8696
**Self-reported manic symptoms**												
Linear regression	0.38 (0.27 to 0.48)	6.78 (14 955)	<.001	14 958	−1.02 (−1.44 to −0.59)	−4.71 (14 858)	<.001	14 868	−0.17 (−0.30 to −0.05)	−2.70 (14 953)	.007	14 958
Co-twin control	0.31 (0.12 to 0.51)	3.14 (104)	.002	105	−1.64 (−2.39 to −0.89)	−4.30 (97)	<.001	105	0.04 (−0.20 to 0.27)	0.30 (102)	.77	105
SCZ GxE	0.17 (0.01 to 0.32)	2.15 (8751)	.03	8797	0.17 (0.01 to 0.32)	2.07 (8699)	.04	8752	0.17 (0.02 to 0.32)	2.19 (8749)	.03	8797
BIP GxE	0.17 (0.01 to 0.32)	2.05 (8751)	.04	8797	0.20 (0.04 to 0.36)	2.39 (8699)	.02	8752	0.17 (0.01 to 0.33)	2.12 (8749)	.03	8797

Abbreviations: BIP GxE, interaction with polygenic score for bipolar I disorder; DF, degrees of freedom; SCZ GxE, interaction with polygenic score for schizophrenia.

^a^ In co-twin control analyses, the number of observations refers to pairs of monozygotic twins where both individuals were included in the analysis. Unadjusted analyses were controlled for sex (except co-twin control analyses, as identical twins have the same sex).

^b^ Substance-specific adjusted analyses controlled for sex (except co-twin control analyses, as identical twins have the same sex) and past use of alcohol, tobacco, cannabis, stimulants, sedatives, opioids, and inhalants.

^c^ Substance-aggregated adjusted analyses controlled for sex (except co-twin control analyses, as identical twins have the same sex), past use of alcohol or tobacco (collapsed into a single variable), and past use of cannabis, stimulants, sedatives, opioids, inhalants, or performance enhancers (collapsed into a single variable).

#### Familial Confounding and Associations Between Psychedelic Use and Self-Reported Psychotic or Manic Symptoms

In unadjusted co-twin control analyses, no significant association was observed between psychedelic use and psychotic symptoms, but manic symptoms were more common in monozygotic twins who had used psychedelics than in their co-twins who had not (β, 0.31; 95% CI, 0.12 to 0.51). In drug-adjusted co-twin control analyses, psychotic symptoms were less common in monozygotic twins who had used psychedelics than in their co-twins who had not used psychedelics (substance-specific adjusted β, −0.89; 95% CI, −1.61 to −0.16 and substance-aggregated adjusted β, −0.24; 95% CI, −0.48 to −0.01). Similar results were found for manic symptoms in substance-specific adjusted analyses (β, −1.64; 95% CI, −2.39 to −0.89), though not in substance-aggregated adjusted analyses.

#### Gene-Environment Interactions on Self-Reported Psychotic or Manic Symptoms

Neither in unadjusted nor drug-adjusted analyses did the association between psychedelic use and psychotic symptoms differ depending on genetic vulnerability to schizophrenia or bipolar I disorder. However, for manic symptoms, there were statistically significant interactions between psychedelic use and genetic vulnerability to schizophrenia (unadjusted β, 0.17; 95% CI, 0.01 to 0.32 and substance-specific adjusted β, 0.17; 95% CI, 0.01 to 0.32; substance-aggregated adjusted β, 0.11; 95% CI, 0.02 to 0.32) or bipolar I disorder (unadjusted β, 0.17; 95% CI, 0.01 to 0.32; substance-specific adjusted β, 0.20; 95% CI, 0.04 to 0.36; substance-aggregated adjusted β, 0.17; 95% CI, 0.01 to 0.33).

Although parent-reported outcomes were not the primary focus of this study, for parent-reported psychotic symptoms at age 15 years, there were statistically significant interactions between psychedelic use and genetic vulnerability to schizophrenia (unadjusted β, 0.09; 95% CI, 0.00 to 0.19; substance-specific adjusted β, 0.11; 95% CI, 0.01 to 0.20; substance-aggregated adjusted β, 0.10; 95% CI, 0.00 to 0.19) (eTable 6 in Supplement 1), in contrast to the nonsignificant findings on self-reported psychotic symptoms at age 15 years.

## Discussion

This cross-sectional study investigated the associations between psychedelic use and self-reported psychotic or manic symptoms in a sample of adolescents using a genetically informative design. When adjusting for substance-specific and substance-aggregated drug use, psychedelic use was associated with fewer psychotic symptoms in both linear regression analyses and co-twin control analyses. In individuals with a higher genetic vulnerability to schizophrenia or bipolar I disorder, psychedelic use was associated with more manic symptoms than in individuals with a lower genetic vulnerability. Taken together, the findings in this study suggest that, after adjusting for other drug use, naturalistic use of psychedelics may be associated with lower rates of psychotic symptoms among adolescents. At the same time, the association between psychedelic use and manic symptoms seems to depend on genetic vulnerability to psychopathology such as schizophrenia or bipolar I disorder.

The associations between psychedelic use and psychotic symptoms were not fully explained by familial confounding (eg, the same genes were associated with both psychedelic use and psychotic symptoms). While these associations should be interpreted with caution, such findings motivate further research into the potential risks and also benefits of psychedelic use among adolescents. The use of co-twin control studies represents a novel research design in psychedelic research that can further inform associations and may be particularly useful when it is not feasible to conduct an experimental study. However, with only cross-sectional data, reverse causality cannot be excluded.

While to our knowledge no modern-day clinical trial using psychedelics has been conducted in individuals diagnosed with schizophrenia or bipolar I disorder, the significant interactions between psychedelic use and genetic vulnerability to schizophrenia or bipolar I disorder on manic symptoms found in this study indicate that mania following psychedelic administration may be more likely in individuals with a personal or family history of schizophrenia or bipolar I disorder. For example, there have been mechanistic conjectures that psychedelics could induce a treatment-emergent affective switch (ie, activation of a manic episode through antidepressant use),^22^ which is one possible explanation of the gene-environment interaction results in this study. However, the evidence remains limited and more studies will be needed to understand the mechanisms underlying the association between psychedelic use and manic symptoms, especially among individuals with genetic vulnerability to schizophrenia or bipolar I disorder.

### Limitations

This study has limitations. First, due to missing data, the total number of responses (yes, no) on past use of psychedelics was higher than the total number of responses on other variables (eg, psychotic symptoms, manic symptoms, and use of other drugs). Second, self-reported manic symptoms were not significantly associated with the polygenic score for bipolar I disorder (eTable 7 in Supplement 1), but the low predictive value is in line with other polygenic score studies on psychiatric traits. When the sample sizes of the genome-wide association study increase, predictive accuracy of the polygenic scores will also increase.^23^ Because of the low predictive value, the results in this study should be interpreted with caution and replication is needed. It is also worth noting that adolescents with a bipolar diagnosis may underreport their manic symptoms,^24^ which could possibly explain these nonsignificant results. Third, there was no information about the context in which the twins had used psychedelics, which could have provided insight into extrapharmacological factors that may be associated with psychotic or manic symptoms following psychedelic use. It would also have been useful to have information on dose and frequency of use, but such data did not exist in this data set. Fourth, the phrasing of some of the items (eg, “It has happened that I have known what another person was thinking although this person wasn’t speaking”) refers to the past in general terms and does not necessarily exclude times of intoxication (ie, acute effects of the drug). It is therefore possible that some of the twins who endorsed these items were referring to a time when they were experiencing the acute effects of psychedelics. Fifth, the self-reported outcomes are susceptible to a range of biases (eg, social desirability bias and recall bias), which may have influenced the results. Sixth, the sample consisted of adolescents and the findings may not be generalizable to other age categories. Seventh, as already mentioned above, the co-twin control design in combination with the nature of the phenotypic data (cross-sectional) cannot exclude reverse causality (eg, it could well be that the twin with fewer psychotic symptoms in the first place is for some reason more likely to consume psychedelics than their co-twin with more psychotic symptoms). Eighth, most participants who reported psychedelic use (99%) also reported use of cannabis, stimulants, sedatives, opioids, inhalants, or performance enhancers, which makes it difficult to disentangle the specific associations with psychedelic use. Ninth, due to the exploratory nature of the study, results were reported without adjustments for multiple tests, which increases the likelihood of observing statistically significant results purely by chance. Taken together, this study has advantages as a population-based naturalistic study that takes genetics into account, but the findings in this study should be interpreted with caution until they have been replicated in future studies.

## Conclusions

The leading guidelines on psychedelic research recommend that individuals with genetic vulnerability to psychotic or bipolar disorders are excluded from participation in clinical trials, but there is a lack of consensus on the risks associated with psychedelic use for these populations, especially among adolescents. In this cross-sectional study of Swedish adolescent twins, we investigated associations between psychedelic use and psychotic or manic symptoms. When adjusting for substance-specific and substance-aggregated drug use, psychedelic use was associated with fewer psychotic symptoms in both linear regression analyses and co-twin control analyses. Psychedelic use was associated with more manic symptoms for individuals with a higher genetic vulnerability to schizophrenia or bipolar I disorder than in individuals with a lower genetic vulnerability, which provides tentative evidence in support of contemporary guidelines on psychedelic research.

In conclusion, this study highlights the potential of genetically informative research designs to delineate the complex interplay between psychedelic use, genetic factors, and psychotic or manic symptoms. Future studies are needed to replicate our findings and extend them to other age groups, ideally with larger samples, longitudinal data, and more objective outcome measures (eg, diagnoses in the health care system).

## Notes

Supplement 1. eTable 1. Twin-Reported Drug Use eTable 2. Twin- and Parent-Reported Psychotic Symptoms at Age 15 eTable 3. Twin- and Parent-Reported Manic Symptoms at Age 15 eTable 4. Variance Inflation Factor in Drug-Adjusted Analyses eTable 5. Model Estimates eTable 6. Descriptive Statistics of Genotyped Twins eTable 7. Associations Between PGS Schizophrenia and Bipolar and Variables at Age 15

Supplement 2. Data sharing statement
