# Supplementary material for: Adolescent Psychedelic Use and Psychotic or Manic Symptoms
Source: JAMA Psychiatry. 2024 Mar 13;81(6):579–85. doi: 10.1001/jamapsychiatry.2024.0047 (PMC10938246; doi:10.1001/jamapsychiatry.2024.0047)
Supplement: Supplement 1. — eTable 1. Twin-Reported Drug Use eTable 2. Twin- and Parent-Reported Psychotic Symptoms at Age 15 eTable 3. Twin- and Parent-Reported Manic Symptoms at Age 15 eTable 4. Variance Inflation Factor in Drug-Adjusted Analyses eTable 5. Model Estimates eTable 6. Descriptive Statistics of Genotyped Twins eTable 7. Associations Between PGS Schizophrenia and Bipolar and Variables at Age 15 [file jamapsychiatry-e240047-s001.pdf]

Supplemental Online Content

Simonsson O, Mosing MA, Osika W, et al. Adolescent psychedelic use and psychotic or manic symptoms. *JAMA Psychiatry*. Published online March 13, 2024. doi:10.1001/jamapsychiatry.2024.0047

eTable 1. Twin-Reported Drug Use

eTable 2. Twin- and Parent-Reported Psychotic Symptoms at Age 15

eTable 3. Twin- and Parent-Reported Manic Symptoms at Age 15

eTable 4. Variance Inflation Factor in Drug-Adjusted Analyses

eTable 5. Model Estimates

eTable 6. Descriptive Statistics of Genotyped Twins

eTable 7. Associations Between PGS Schizophrenia and Bipolar and Variables at Age 15

This supplemental material has been provided by the authors to give readers additional information about their work.

© 2024 Simonsson O et al. *JAMA Psychiatry.*

eTable 1. Twin-Reported Drug Use			
Items	Response Options	Category 1	Category 2
LSD, "acid"	*Never tried*	*Psychedelics*	*Psychedelics*
LSD, "acid"	*At least once*	*Psychedelics*	*Psychedelics*
LSD, "acid"	*At least once in the last year*	*Psychedelics*	*Psychedelics*
LSD, "acid"	*At least once in the last month*	*Psychedelics*	*Psychedelics*
Magic mushrooms, Psilocybin	*Never tried*	*Psychedelics*	*Psychedelics*
Magic mushrooms, Psilocybin	*At least once*	*Psychedelics*	*Psychedelics*
Magic mushrooms, Psilocybin	*At least once in the last year*	*Psychedelics*	*Psychedelics*
Magic mushrooms, Psilocybin	*At least once in the last month*	*Psychedelics*	*Psychedelics*
Beer (excluding lightbeer), or strong cider	*Never tried*	*Alcohol*	*Alcohol or tobacco*
Beer (excluding lightbeer), or strong cider	*At least once*	*Alcohol*	*Alcohol or tobacco*
Beer (excluding lightbeer), or strong cider	*At least once in the last year*	*Alcohol*	*Alcohol or tobacco*
Beer (excluding lightbeer), or strong cider	*At least once in the last month*	*Alcohol*	*Alcohol or tobacco*
Wine, alcopops, shots, drinks or hard liquor/schnapps (e.g. brandy, moonshine, vodka, gin, cognac, whisky, liqueur, punch)	*Never tried*	*Alcohol*	*Alcohol or tobacco*
Wine, alcopops, shots, drinks or hard liquor/schnapps (e.g. brandy, moonshine, vodka, gin, cognac, whisky, liqueur, punch)	*At least once*	*Alcohol*	*Alcohol or tobacco*
Wine, alcopops, shots, drinks or hard liquor/schnapps (e.g. brandy, moonshine, vodka, gin, cognac, whisky, liqueur, punch)	*At least once in the last year*	*Alcohol*	*Alcohol or tobacco*
Wine, alcopops, shots, drinks or hard liquor/schnapps (e.g. brandy, moonshine, vodka, gin, cognac, whisky, liqueur, punch)	*At least once in the last month*	*Alcohol*	*Alcohol or tobacco*
Do you smoke cigarettes?	*No, I have never smoked*	*Tobacco*	*Alcohol or tobacco*
Do you smoke cigarettes?	*No, I have only tried*	*Tobacco*	*Alcohol or tobacco*
Do you smoke cigarettes?	*No, I quit*	*Tobacco*	*Alcohol or tobacco*
Do you smoke cigarettes?	*Yes, but only sometimes*	*Tobacco*	*Alcohol or tobacco*
Do you smoke cigarettes?	*Yes, almost every day*	*Tobacco*	*Alcohol or tobacco*
Do you use snuff?	*No, I have never smoked*	*Tobacco*	*Alcohol or tobacco*
Do you use snuff?	*No, I have only tried*	*Tobacco*	*Alcohol or tobacco*
Do you use snuff?	*No, I quit*	*Tobacco*	*Alcohol or tobacco*
Do you use snuff?	*Yes, but only sometimes*	*Tobacco*	*Alcohol or tobacco*
Do you use snuff?	*Yes, almost every day*	*Tobacco*	*Alcohol or tobacco*
Cannabis, marijuana or hashish	*Never tried*	*Cannabis*	*Other drugs*
Cannabis, marijuana or hashish	*At least once*	*Cannabis*	*Other drugs*
Cannabis, marijuana or hashish	*At least once in the last year*	*Cannabis*	*Other drugs*
Cannabis, marijuana or hashish	*At least once in the last month*	*Cannabis*	*Other drugs*
Amphetamine, khat	*Never tried*	*Stimulants*	*Other drugs*

© 2024 Simonsson O et al. *JAMA Psychiatry.*

Amphetamine, khat	*At least once*	*Stimulants*	*Other drugs*
Amphetamine, khat	*At least once in the last year*	*Stimulants*	*Other drugs*
Amphetamine, khat	*At least once in the last month*	*Stimulants*	*Other drugs*
Cocaine, crack	*Never tried*	*Stimulants*	*Other drugs*
Cocaine, crack	*At least once*	*Stimulants*	*Other drugs*
Cocaine, crack	*At least once in the last year*	*Stimulants*	*Other drugs*
Cocaine, crack	*At least once in the last month*	*Stimulants*	*Other drugs*
Ecstasy	*Never tried*	*Stimulants*	*Other drugs*
Ecstasy	*At least once*	*Stimulants*	*Other drugs*
Ecstasy	*At least once in the last year*	*Stimulants*	*Other drugs*
Ecstasy	*At least once in the last month*	*Stimulants*	*Other drugs*
Sleeping pills, tranquilizers (te.g. Rohypnol, Nitrazepam, Sobril, Stesolid, Valium, Xanor, Imovane, Stilnoct)	*Never tried*	*Sedatives*	*Other drugs*
Sleeping pills, tranquilizers (te.g. Rohypnol, Nitrazepam, Sobril, Stesolid, Valium, Xanor, Imovane, Stilnoct)	*At least once*	*Sedatives*	*Other drugs*
Sleeping pills, tranquilizers (te.g. Rohypnol, Nitrazepam, Sobril, Stesolid, Valium, Xanor, Imovane, Stilnoct)	*At least once in the last year*	*Sedatives*	*Other drugs*
Sleeping pills, tranquilizers (te.g. Rohypnol, Nitrazepam, Sobril, Stesolid, Valium, Xanor, Imovane, Stilnoct)	*At least once in the last month*	*Sedatives*	*Other drugs*
Heroin, smoke heroin or opium	*Never tried*	*Opioids*	*Other drugs*
Heroin, smoke heroin or opium	*At least once*	*Opioids*	*Other drugs*
Heroin, smoke heroin or opium	*At least once in the last year*	*Opioids*	*Other drugs*
Heroin, smoke heroin or opium	*At least once in the last month*	*Opioids*	*Other drugs*
Morphine, dolcontin, ketogan, ketodur, methadone	*Never tried*	*Opioids*	*Other drugs*
Morphine, dolcontin, ketogan, ketodur, methadone	*At least once*	*Opioids*	*Other drugs*
Morphine, dolcontin, ketogan, ketodur, methadone	*At least once in the last year*	*Opioids*	*Other drugs*
Morphine, dolcontin, ketogan, ketodur, methadone	*At least once in the last month*	*Opioids*	*Other drugs*
Painkillers (te.g. Distalgesic, Citodon, Kodein, Treo Comp)	*Never tried*	*Opioids*	*Other drugs*
Painkillers (te.g. Distalgesic, Citodon, Kodein, Treo Comp)	*At least once*	*Opioids*	*Other drugs*
Painkillers (te.g. Distalgesic, Citodon, Kodein, Treo Comp)	*At least once in the last year*	*Opioids*	*Other drugs*
Painkillers (te.g. Distalgesic, Citodon, Kodein, Treo Comp)	*At least once in the last month*	*Opioids*	*Other drugs*
Sniffed lighter gas, tri, gasoline/fuel or glue	*Never tried*	*Inhalants*	*Other drugs*
Sniffed lighter gas, tri, gasoline/fuel or glue	*At least once*	*Inhalants*	*Other drugs*
Sniffed lighter gas, tri, gasoline/fuel or glue	*At least once in the last year*	*Inhalants*	*Other drugs*
Sniffed lighter gas, tri, gasoline/fuel or glue	*At least once in the last month*	*Inhalants*	*Other drugs*
GHB	*Never tried*	*Performance-enhancers*	*Other drugs*
GHB	*At least once*	*Performance-enhancers*	*Other drugs*

© 2024 Simonsson O et al. *JAMA Psychiatry.*

GHB	*At least once in the last year*	*Performance-enhancers*	*Other drugs*
GHB	*At least once in the last month*	*Performance-enhancers*	*Other drugs*
Anabolic Steroids	*Never tried*	*Performance-enhancers*	*Other drugs*
Anabolic Steroids	*At least once*	*Performance-enhancers*	*Other drugs*
Anabolic Steroids	*At least once in the last year*	*Performance-enhancers*	*Other drugs*
Anabolic Steroids	*At least once in the last month*	*Performance-enhancers*	*Other drugs*
Note: Respondents reporting never having used any of the drugs within each category were coded as 0, whereas those who reported past use of any of the drugs within each category were coded as 1.			

© 2024 Simonsson O et al. *JAMA Psychiatry.*

eTable 2. Twin- and Parent-Reported Psychotic Symptoms at Age 15
Twin-reported psychotic symptoms
Sometime I thought that I was being followed or spied upon.
Other people have read my thoughts.
Sometime I thought I was being sent special messages through the television.
I have special powers that other people don't have.
Sometime I felt that I was under the control of some special power.
It has happened that I have known what another person was thinking although this person wasn't speaking.
Sometime I have seen something or someone that other people couldn't see.
Parent-reported psychotic symptoms
Some people believe that their thoughts can be read by another person. Has your child ever thought that other people have read his/her thoughts?
Has he/she ever thought he/she was being sent special messages through the television?
Has he/she ever thought that he/she was being followed or spied upon?
Has he/she ever felt that he/she was under the control of some special power?
Has he/she ever known what another person was thinking although this person wasn't speaking?
Has he/she ever believed he/she had any special powers that other people don't have?
Has he/she ever seen something or someone that other people couldn't see?
The response options for each item (apart from "Don't know" and "Don't want to answer") was the following: "No, never"; "Maybe"; "Definitely".

© 2024 Simonsson O et al. *JAMA Psychiatry.*

eTable 3. Twin- and Parent-Reported Manic Symptoms at Age 15
Twin-reported manic symptoms
Does it happen that you…
Have periods of feeling super happy for hours or days at a time, extremely wound up and excited, such as feeling "on top of the world"?
Feel irritable, cranky, or mad for hours or days at a time?
Believe that I have unrealistic abilities or powers that are unusual, and may try to act upon them, which causes trouble?
Need less sleep than usual; yet do not feel tired the next day?
Have periods of too much energy?
Have periods of racing thoughts that my mind cannot slow down, and it seems that my mouth cannot keep up with my mind?
Talk so fast that I jump from topic to topic?
Behave in a sexually inappropriate way (e.g., talking dirty, exposing yourself, playing with your private parts, masturbating, making sex phone calls, humping on dogs, playing sex games, touching others sexually)?
Have rage attacks, intense and prolonged temper tantrums?
Hear voices that nobody else can hear?
Parent-reported manic symptoms
Does your child…
Have periods of feeling super happy for hours or days at a time, extremely wound up and excited, such as feeling "on top of the world"?
Feel irritable, cranky, or mad for hours or days at a time?
Believe that he or she has unrealistic abilities or powers that are unusual, and may try to act upon them, which causes trouble?
Need less sleep than usual; yet does not feel tired the next day?
Have periods of too much energy?
Have periods of racing thoughts that his or her mind cannot slow down, and it seems that your child's mouth cannot keep up with his or her mind?
Talk so fast that he or she jumps from topic to topic?
Behave in a sexually inappropriate way (e.g., talks dirty, exposing, playing with private parts, masturbating, making sex phone calls, humping on dogs, playing sex games, touches others sexually)?
Have rage attacks, intense and prolonged temper tantrums?
Hear voices that nobody else can hear?
The response options for each item (apart from "Don't know" and "Don't want to answer") was the following: "Never/Rarely"; "Sometimes"; "Often"; "Very Often".

© 2024 Simonsson O et al. *JAMA Psychiatry.*

eTable 4. Variance Inflation Factor in Drug-Adjusted Analyses		
	Self-Reported Psychotic Symptoms (Age 15)	
	Substance-Specific Adjusted Analyses (with Performance-Enhancers)	Substance-Specific Adjusted Analyses (without Performance-Enhancers)
Past Use of Psychedelics	16.54	9.29
Past Use of Alcohol	1.40	1.40
Past Use of Tobacco	1.37	1.37
Past Use of Cannabis	2.74	2.74
Past Use of Stimulants	8.93	8.71
Past Use of Sedatives	3.65	3.49
Past Use of Opioids	1.89	1.89
Past Use of Inhalants	2.58	2.52
Past Use of Performance-Enhancers	14.38	…
Sex	1.01	1.01
	Substance-Aggregated Adjusted Analyses	…
Past Use of Psychedelics	1.37	…
Past Use of Alcohol or Tobacco	1.07	…
Past Use of Other Drugs	1.42	…
Sex	1.00	…
	Self-Reported Manic Symptoms (Age 15)	
	Substance-Specific Adjusted Analyses (with Performance-Enhancers)	Substance-Specific Adjusted Analyses (without Performance-Enhancers)
Past Use of Psychedelics	16.98	9.77
Past Use of Alcohol	1.40	1.40
Past Use of Tobacco	1.38	1.37
Past Use of Cannabis	2.70	2.70
Past Use of Stimulants	9.35	9.09
Past Use of Sedatives	3.65	3.49
Past Use of Opioids	1.87	1.87
Past Use of Inhalants	2.53	2.46
Past Use of Performance-Enhancers	14.61	…
Sex	1.00	1.00
	Substance-Aggregated Adjusted Analyses	…
Past Use of Psychedelics	1.37	…
Past Use of Alcohol or Tobacco	1.07	…
Past Use of Other Drugs	1.41	…

© 2024 Simonsson O et al. *JAMA Psychiatry.*

Sex	1.00	…
This table presents variance inflation factor in (Model 1) drug-adjusted regression models on primary outcome variables. Note that substance-specific adjusted analyses (with performance-enhancers) analyses were dropped due to high variance inflation factor (>10) for past use of performance-enhancers.		

© 2024 Simonsson O et al. *JAMA Psychiatry.*

eTable 5. Descriptive Statistics of Genotyped Twins			
	**Genotyped twins**		
	Yes (n = 9,426)	No (n = 6,829)	
	n (%)	n (%)	*p*
**Past Use of Psychedelics**			.097
Yes	295 (3)	246 (4)	
No	9,131 (97)	6,583 (96)	
**Past Use of Alcohol**			.582
Yes	4,241 (45)	3,102 (45)	
No	5,183 (55)	3,725 (55)	
**Past Use of Tobacco**			<.001
Yes	2,527 (27)	1,999 (29)	
No	6,887 (73)	4,808 (71)	
**Past Use of Cannabis**			.001
Yes	463 (5)	420 (6)	
No	8,957 (95)	6,397 (94)	
**Past Use of Stimulants**			.027
Yes	315 (3)	273 (4)	
No	9,111 (97)	6,556 (96)	
**Past Use of Sedatives**			.031
Yes	394 (4)	334 (5)	
No	9,011 (96)	6,482 (95)	
**Past Use of Opioids**			.005
Yes	645 (7)	546 (8)	
No	8,781 (93)	6,283 (92)	
**Past Use of Inhalants**			.118
Yes	467 (5)	376 (6)	
No	8,946 (95)	6,444 (94)	
**Past Use of Performance-Enhancers**			.118
Yes	282 (3)	234 (3)	
No	9,143 (97)	6,593 (97)	
**Sex**			.031
Female	5,087 (54)	3,802 (56)	
Male	4,339 (46)	3,027 (44)	

© 2024 Simonsson O et al. *JAMA Psychiatry.*

Note: n = number of cases; percentages are presented within brackets and are rounded to the closest integer. Due to missing data, total numbers for each category may not add up to total number of responses on genotyped twins (N = 16,255). Pearson's chi-squared tests were used to examine the characteristics of genotyped twins versus non-genotyped twins.

© 2024 Simonsson O et al. *JAMA Psychiatry.*

eTable 6. Model Estimates												
Parent-Reported Psychotic Symptoms (Age 15)												
Model	Unadjusted Analyses				Substance-Specific Adjusted Analyses				Substance-Aggregated Adjusted Analyses			
	β (95% CI)	*t*	*p*	*n*	*β* (95% CI)	*t*	*p*	*n*	*β* (95% CI)	*t*	*p*	*n*
Linear regression	0.05 (-0.06 – 0.15)	0.84	0.398	11,966	-0.26 (-0.61 – 0.10)	-1.40	0.161	11,892	-0.13 (-0.26 – 0.00)	-1.90	0.057	11,966
Co-twin control	0.05 (-0.10 – 0.21)	0.68	0.496	87	-0.30 (-0.94 – -0.34)	-0.92	0.356	86	0.01 (-0.18 – 0.20)	0.10	0.923	87
SCZ GxE	0.09 (0.00 – 0.19)	1.99	0.047	7,787	0.11 (0.01 – 0.20)	2.23	0.026	7,744	0.10 (0.00 – 0.19)	2.02	0.043	7,787
BIP GxE	0.01 (-0.09 – 0.12)	0.25	0.800	7,787	0.01 (-0.11 – 0.12)	0.13	0.897	7,744	0.01 (-0.10 – 0.12)	0.25	0.805	7,787
Self-Reported Psychotic Symptoms (Age 18)												
Model	Unadjusted Analyses				Substance-Specific Adjusted Analyses				Substance-Aggregated Adjusted Analyses			
	β (95% CI)	*t*	*p*	*n*	*β* (95% CI)	*t*	*p*	*n*	*β* (95% CI)	*t*	*p*	*n*
Linear regression	0.13 (0.01 – 0.25)	2.08	0.037	8,296	-0.92 (-1.54 – -0.30)	-2.92	0.003	8,249	-0.25 (-0.41 – -0.09)	-3.04	0.002	8,296
Co-twin control	0.28 (0.00 – 0.56)	1.97	0.049	52	0.10 (-1.05 – 1.24)	0.16	0.870	51	0.11 (-0.23 – 0.45)	0.66	0.510	52
SCZ GxE	-0.02 (-0.18 – 0.13)	-0.31	0.757	5,405	-0.01 (-0.17 – 0.15)	-0.12	0.907	5,380	-0.02 (-0.17 – 0.14)	-0.22	0.824	5,405
BIP GxE	-0.03 (-0.21 – 0.16)	-0.28	0.780	5,405	-0.03 (-0.22 – 0.16)	-0.34	0.734	5,380	-0.02 (-0.21 – 0.16)	-0.26	0.793	5,405
Parent-Reported Psychotic Symptoms (Age 18)												
Model	Unadjusted Analyses				Substance-Specific Adjusted Analyses				Substance-Aggregated Adjusted Analyses			
	β (95% CI)	*t*	*p*	*n*	*β* (95% CI)	*t*	*p*	*n*	*β* (95% CI)	*t*	*p*	*n*
Linear regression	-0.04 (-0.15 – 0.08)	-0.64	0.521	6,964	-0.76 (-1.30 – -0.22)	-2.78	0.005	6,922	-0.21 (-0.37 – -0.05)	-2.52	0.012	6,964
Co-twin control	0.05 (-0.16 – 0.26)	0.50	0.619	47	1.24 (0.32 – 2.17)	2.64	0.008	46	0.17 (-0.09 – 0.42)	1.27	0.205	47
SCZ GxE	0.11 (-0.10 – 0.32)	1.03	0.304	4,949	0.12 (-0.09 – 0.33)	1.09	0.276	4,920	0.11 (-0.10 – 0.33)	1.05	0.294	4,949
BIP GxE	-0.08 (-0.21 – 0.05)	-1.21	0.225	4,949	-0.12 (-0.25 – 0.01)	-1.76	0.079	4,920	-0.08 (-0.21 – 0.05)	-1.21	0.226	4,949
Self-Reported Psychotic Symptoms (Age 24)												
Model	Unadjusted Analyses				Substance-Specific Adjusted Analyses				Substance-Aggregated Adjusted Analyses			
	β (95% CI)	*t*	*p*	*n*	*β* (95% CI)	*t*	*p*	*n*	*β* (95% CI)	*t*	*p*	*n*
Linear regression	0.03 (-0.17 – 0.22)	0.27	0.789	3,816	-0.62 (-1.48 – 0.24)	-1.41	0.158	3,792	-0.34 (-0.59 – -0.10)	-2.75	0.006	3,816
Co-twin control	0.16 (-0.30 – 0.62)	0.69	0.493	18	0.26 (-1.36 – 1.89)	0.32	0.751	18	0.11 (-0.41 – 0.62)	0.40	0.689	18
SCZ GxE	-0.18 (-0.50 – 0.14)	-1.10	0.271	2,442	-0.12 (-0.46 – 0.21)	-0.73	0.468	2,430	-0.18 (-0.50 – 0.15)	-1.07	0.284	2,442
BIP GxE	0.12 (-0.12 – 0.37)	0.98	0.326	2,442	0.18 (-0.08 – 0.44)	1.34	0.180	2,430	0.13 (-0.12 – 0.38)	0.99	0.321	2,442
Parent-Reported Manic Symptoms (Age 15)												
Model	Unadjusted Analyses				Substance-Specific Adjusted Analyses				Substance-Aggregated Adjusted Analyses			
	β (95% CI)	*t*	*p*	*n*	*β* (95% CI)	*t*	*p*	*n*	*β* (95% CI)	*t*	*p*	*n*
Linear regression	0.15 (0.04 – 0.26)	2.65	0.008	12,091	-0.27 (-0.66 – 0.13)	-1.33	0.184	12,014	-0.07 (-0.21 – 0.06)	-1.10	0.269	12,091
Co-twin control	-0.02 (-0.18 – 0.13)	-0.28	0.779	87	-0.29 (-0.96 – 0.38)	-0.85	0.394	86	-0.06 (-0.25 – 0.12)	-0.67	0.504	87
SCZ GxE	0.01 (-0.12 – 0.15)	0.19	0.848	7,867	0.01 (-0.13 – 0.15)	0.13	0.896	7,824	0.01 (-0.12 – 0.15)	0.21	0.832	7,867

© 2024 Simonsson O et al. *JAMA Psychiatry.*

BIP GxE	0.09 (-0.04 – 0.22)	1.42	0.156	7,867	0.12 (-0.01 – 0.25)	1.83	0.067	7,824	0.09 (-0.03 – 0.22)	1.43	0.154	7,867
Self-Reported Manic Symptoms (Age 18)												
Model	Unadjusted Analyses				Substance-Specific Adjusted Analyses				Substance-Aggregated Adjusted Analyses			
	β (95% CI)	*t*	*p*	*n*	*β* (95% CI)	*t*	*p*	*n*	*β* (95% CI)	*t*	*p*	*n*
Linear regression	0.14 (0.00 – 0.28)	1.93	0.053	8,706	-0.49 (-1.11 – 0.14)	-1.52	0.130	8,650	-0.11 (-0.28 – 0.05)	-1.33	0.185	8,706
Co-twin control	0.04 (-0.24 – 0.32)	0.26	0.794	54	-0.41 (-1.58 – 0.75)	-0.70	0.486	53	-0.05 (-0.39 – 0.29)	-0.30	0.762	54
SCZ GxE	0.05 (-0.14 – 0.24)	0.55	0.586	5,598	0.06 (-0.13 – 0.25)	0.64	0.519	5,571	0.05 (-0.14 – 0.24)	0.56	0.576	5,598
BIP GxE	0.05 (-0.20 – 0.30)	0.41	0.683	5,598	0.07 (-0.17 – 0.32)	0.57	0.570	5,571	0.05 (-0.20 – 0.30)	0.41	0.680	5,598

Unadjusted Analyses = controlling for sex (except co-twin control analyses as identical twins have the same sex). Substance-Specific Adjusted Analyses = controlling for sex (except co-twin control analyses as identical twins have the same sex) and past use of alcohol, tobacco, cannabis, stimulants, sedatives, opioids, and inhalants. Substance-Aggregated Adjusted Analyses = controlling for sex (except co-twin control analyses as identical twins have the same sex), past use of alcohol or tobacco (collapsed into a single variable), and past use of cannabis, stimulants, sedatives, opioids, inhalants, or performance-enhancers (collapsed into a single variable). SCZ GxE = Interaction with PGS for schizophrenia; BIP GxE = Interaction with PGS for bipolar I disorder.

© 2024 Simonsson O et al. *JAMA Psychiatry.*

eTable 7. Associations Between PGS Schizophrenia and Bipolar I Disorder and Variables at Age 15				
Past Use of Psychedelics				
Model	Unadjusted Analyses			
	β (95% CI)	*z*	*p*	*n*
SCZ	0.04 (-0.08 – 0.15)	0.58	0.560	9,426
BIP	0.02 (-0.10 – 0.13)	0.28	0.783	9,426
Past Use of Alcohol				
Model	Unadjusted Analyses			
	β (95% CI)	*z*	*p*	*n*
SCZ	0.04 (-0.01 – 0.08)	1.50	0.135	9,429
BIP	0.06 (0.02 – 0.11)	2.58	0.010	9,429
Past Use of Tobacco				
Model	Unadjusted Analyses			
	β (95% CI)	*z*	*p*	*n*
SCZ	0.10 (0.04 – 0.15)	3.57	<0.001	9,429
BIP	0.11 (0.06 – 0.16)	3.96	<0.001	9,429
Past Use of Cannabis				
Model	Unadjusted Analyses			
	β (95% CI)	*z*	*p*	*n*
SCZ	0.07 (-0.03 – 0.17)	1.30	0.194	9,420
BIP	0.10 (0.01 – 0.20)	2.08	0.037	9,420
Past Use of Stimulants				
Model	Unadjusted Analyses			
	β (95% CI)	*z*	*p*	*n*
SCZ	0.03 (-0.08 – 0.15)	0.60	0.549	9,426
BIP	0.05 (-0.07 – 0.16)	0.78	0.435	9,426
Past Use of Sedatives				
Model	Unadjusted Analyses			
	β (95% CI)	*z*	*p*	*n*
SCZ	0.09 (-0.01 – 0.20)	1.80	0.071	9,405
BIP	0.08 (-0.03 – 0.18)	1.44	0.149	9,405
Past Use of Opioids				
Model	Unadjusted Analyses			
	β (95% CI)	*z*	*p*	*n*
SCZ	0.04 (-0.04 – 0.12)	1.05	0.292	9,426
BIP	0.01 (-0.08 – 0.09)	0.18	0.857	9,426

© 2024 Simonsson O et al. *JAMA Psychiatry.*

Past Use of Inhalants				
Model	Unadjusted Analyses			
	β (95% CI)	*z*	*p*	*n*
SCZ	0.03 (-0.07 – 0.13)	0.58	0.564	9,413
BIP	0.04 (-0.05 – 0.13)	0.81	0.416	9,413
Past Use of Performance-Enhancers				
Model	Unadjusted Analyses			
	β (95% CI)	*z*	*p*	*n*
SCZ	0.02 (-0.10 – 0.14)	0.36	0.716	9,425
BIP	0.00 (-0.11 – 0.12)	0.07	0.941	9,425
Self-Reported Psychotic Symptoms (Age 15)				
Model	Unadjusted Analyses			
	β (95% CI)	*t*	*p*	*n*
SCZ	0.07 (0.04 – 0.09)	5.42	<0.001	8,766
BIP	0.05 (0.02 – 0.07)	3.70	<0.001	8,766
Parent-Reported Psychotic Symptoms (Age 15)				
Model	Unadjusted Analyses			
	β (95% CI)	*t*	*p*	*n*
SCZ	0.02 (-0.01 – 0.04)	1.29	0.195	8,448
BIP	-0.01 (-0.03 – 0.01)	-0.78	0.434	8,448
Self-Reported Manic Symptoms (Age 15)				
Model	Unadjusted Analyses			
	β (95% CI)	*t*	*p*	*n*
SCZ	0.02 (-0.01 – 0.04)	1.52	0.128	8,827
BIP	0.02 (0.00 – 0.04)	1.71	0.088	8,827
Parent-Reported Manic Symptoms (Age 15)				
Model	Unadjusted Analyses			
	β (95% CI)	*t*	*p*	*n*
SCZ	0.03 (0.00 – 0.05)	2.05	0.040	8,540
BIP	0.02 (0.00 – 0.05)	1.62	0.105	8,540
Unadjusted Analyses = controlling for sex. SCZ = PGS for schizophrenia as independent variable; BIP = PGS for bipolar I disorder as independent variable. None of the models controlled for other drug use.				

© 2024 Simonsson O et al. *JAMA Psychiatry.*
