# Supplementary material for: Adolescent Psychedelic Use and Psychotic or Manic Symptoms
Source: JAMA Psychiatry. 2024 Mar 13;81(6):579–85. doi: 10.1001/jamapsychiatry.2024.0047 (PMC10938246; doi:10.1001/jamapsychiatry.2024.0047)
Supplement: Supplement 2. — Data sharing statement [file jamapsychiatry-e240047-s002.pdf]

Data Sharing Statement

Simonsson. Adolescent Psychedelic Use and Psychotic or Manic Symptoms. *JAMA Psychiatry*. Published March 13, 2024. doi:10.1001/jamapsychiatry.2024.0047

Data
Data available: No
